# Validation of a Dynamic Risk Prediction Model Incorporating Prior Mammograms in a Diverse Population

**DOI:** 10.1001/jamanetworkopen.2025.12681

**Published:** 2025-06-06

**Authors:** Shu Jiang, Debbie L. Bennett, Graham A. Colditz

**Affiliations:** 1 Division of Public Health Sciences, Department of Surgery, Washington University School of Medicine in St Louis, St Louis, Missouri; 2 Department of Radiology, Washington University School of Medicine in St Louis, St Louis, Missouri

## Abstract

**Importance:**

For breast cancer risk prediction to be clinically useful, it must be accurate and applicable to diverse groups of women across multiple settings.

**Objective:**

To examine whether a dynamic risk prediction model incorporating prior mammograms, previously validated in Black and White women, could predict future risk of breast cancer across a racially and ethnically diverse population in a population-based screening program.

**Design, Setting, and Participants:**

This prognostic study included women aged 40 to 74 years with 1 or more screening mammograms drawn from the British Columbia Breast Screening Program from January 1, 2013, to December 31, 2019, with follow-up via linkage to the British Columbia Cancer Registry through June 2023. This provincial, organized screening program offers screening mammography with full field digital mammography (FFDM) every 2 years. Data were analyzed from May to August 2024.

**Exposure:**

FFDM-based, artificial intelligence–generated mammogram risk score (MRS), including up to 4 years of prior mammograms.

**Main Outcomes and Measures:**

The primary outcomes were 5-year risk of breast cancer (measured with the area under the receiver operating characteristic curve [AUROC]) and absolute risk of breast cancer calibrated to the US Surveillance, Epidemiology, and End Results incidence rates.

**Results:**

Among 206 929 women (mean [SD] age, 56.1 [9.7] years; of 118 093 with data on race, there were 34 266 East Asian; 1946 Indigenous; 6116 South Asian; and 66 742 White women), there were 4168 pathology-confirmed incident breast cancers diagnosed through June 2023. Mean (SD) follow-up time was 5.3 (3.0) years. Using up to 4 years of prior mammogram images in addition to the most current mammogram, a 5-year AUROC of 0.78 (95% CI, 0.77-0.80) was obtained based on analysis of images alone. Performance was consistent across subgroups defined by race and ethnicity in East Asian (AUROC, 0.77; 95% CI, 0.75-0.79), Indigenous (AUROC, 0.77; 95% CI 0.71-0.83), and South Asian (AUROC, 0.75; 95% CI 0.71-0.79) women. Stratification by age gave a 5-year AUROC of 0.76 (95% CI, 0.74-0.78) for women aged 50 years or younger and 0.80 (95% CI, 0.78-0.82) for women older than 50 years. There were 18 839 participants (9.0%) with a 5-year risk greater than 3%, and the positive predictive value was 4.9% with an incidence of 11.8 per 1000 person-years.

**Conclusions and Relevance:**

A dynamic MRS generated from both current and prior mammograms showed robust performance across diverse racial and ethnic populations in a province-wide screening program starting from age 40 years, reflecting improved accuracy for racially and ethnically diverse populations.

## Key Points

**Question:**

Can an artificial intelligence–based breast cancer risk prediction model incorporating prior mammograms be accurate and applicable across diverse groups of women and in multiple settings for estimating future risk of breast cancer?

**Findings:**

In this prognostic study of 206 929 women aged 40 to 74 years participating in the British Columbia Breast Screening Program, the 5-year area under the receiver operating characteristic curve from the model incorporating prior screening images was consistent across racial and ethnic groups, showing high discriminatory performance and improved prediction when compared with use of a single screening mammogram time point.

**Meaning:**

These findings suggest that this model is accurate and generalizable across a racially and ethnically diverse population and support greater use of available mammogram data from an individual’s current and previous images to improve personal risk assessment.

## Teaser

This prognostic study uses a risk prediction model incorporating prior mammograms to predict future risk of breast cancer across a diverse population in British Columbia, Canada.

## Introduction

Breast cancer screening programs have historically relied on population-level modeling for guideline development. Definitions of women at high risk and average risk for development of breast cancer use clinical and epidemiological risk factors such as known genetic variants and family history, among others. The addition of mandated reporting of breast density highlights the need for individual decision-making for screening.^1^ Current methods of risk estimation are not accurate enough to guide individual decisions regarding risk reduction strategies in women at high-risk of breast cancer^2,3^ and to guide decisions regarding appropriate screening regimens.^4^

Although current artificial intelligence (AI)–based models using mammograms from a single point in time outperform traditional clinical risk assessment models, their accuracy remains insufficient for making personalized screening decisions. A comprehensive comparison of risk prediction models from Kaiser Permanente^5^ reported that combining clinical risk factors with AI analysis of a single mammogram achieved a 5-year area under the receiver operating characteristic curve (AUROC) of 0.63 to 0.67. In contrast, incorporating both current and prior mammograms captures temporal changes, such as those in breast density, that are significantly associated with long-term risk.^6,7,8^ Leveraging a dynamic risk prediction model—which fully integrates each woman’s mammogram image history to predict future risk—was found to substantially improve 5-year prediction, achieving an AUROC of 0.81 in an external validation.^9,10^

For use in routine screening services, models need to be broadly applicable to women in all racial and ethnic groups. Previous models are largely developed and validated on White women and compared with small subsets of women of other races and ethnicities.^11^ Furthermore, performance of previous AI models drops significantly when validated in racially and ethnically diverse populations of women.^5,11^ While our dynamic risk prediction model was validated in Black women with robust performance,^10^ other racial and ethnic groups, such as Asian women, were underrepresented.

When including changes in mammograms over time, models also need to be robust in both opportunistic and routine population-based screening programs. The dynamic risk prediction model was developed at Washington University where data included multiple years of digital mammogram images from women in an American College of Radiology–accredited opportunistic screening service.^12^ The outcome was pathology-confirmed incident breast cancers diagnosed from 2010 thorough 2020. The development and initial validation populations included Black and White women. Evaluation of model performance in routine organized screening programs such as those provided by health agencies in Europe, Canada, and Australia is a necessary step toward broader application of personalized detection and prevention of breast cancer.^4,13^

Here we evaluate our dynamic risk prediction model^9,10^ in a province-wide screening program in British Columbia, Canada. Mammographic breast screening starts at age 40 years and is provided every other year, consistent with recently updated US guidelines from the US Preventive Services Task Force (USPSTF).^14^

## Methods

### British Columbia Breast Screening Cohort

The external validation dataset for this prognostic study was drawn from the British Columbia Breast Screening Program, Canada’s first population-based screening mammography program.^15^ We obtained agreement with British Columbia Cancer and with ethics approval through the University of British Columbia and British Columbia Cancer research ethics board to study performance of breast cancer risk prediction based on whole mammogram images and followed the Transparent Reporting of a Multivariable Prediction Model for Individual Prognosis or Diagnosis (TRIPOD) reporting guideline for prediction models. Mammogram images were assembled from 7 sites in British Columbia, including 2 mobile clinics, that routinely provide central storage of screening images for quality control and program evaluation.^16^ Informed consent was not required because deidentified data were accessed through a research ethics board–approved protocol to achieve the aims of the approved proposal.

This cohort includes prospective data from 208 288 women screened with full-field digital mammogram (FFDM) from January 1, 2013, to December 31, 2019, and linked to the provincial tumor registry by provincial staff blind to mammography features for incident breast cancer diagnosed through June 20, 2023. Maximum follow-up from initial screening was 10 years. Those who received a diagnosis of breast cancer within the first 6 months of entry to cohort and those with a diagnosis prior to entry to cohort were excluded (eFigure 1 in Supplement 1). Risk factor information including population group was collected at screening centers by self-report^17^ and used to evaluate performance across participating populations.^18^ Asymptomatic women aged 40 to 74 years with no personal history of breast cancer are eligible for screening mammography at 1 of 36 fixed sites and 3 mobile screening sites.^15,19,20,21^ The majority of screening mammograms (95%) are performed on Hologic machines, while 5% are performed on General Electric machines.

### Statistical Analysis

**Figure 1.**
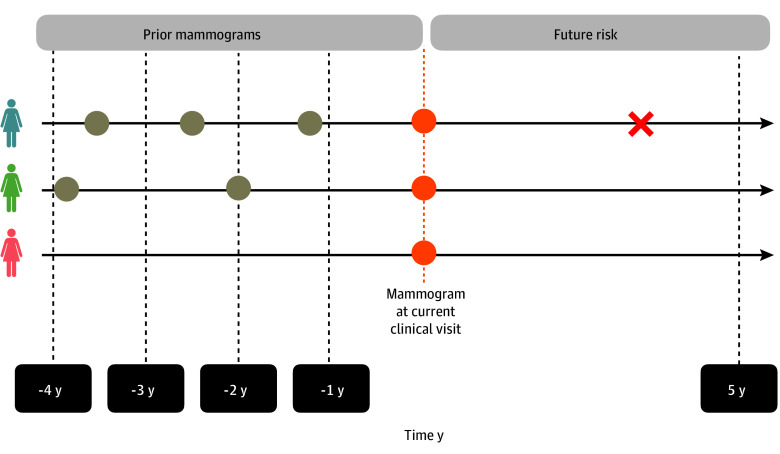
Dynamic Risk Prediction Using Prior Mammograms With Current Visit to Predict a 5-Year Future Risk of Breast Cancer The model is compatible with women with a current mammogram only (red) as well as those with prior history of mammograms accompanied with various visit intervals (green and blue). Orange dots denote index mammogram, and brown dots denote prior mammograms. The red X indicates breast cancer.

We applied the previously validated dynamic risk prediction model to predict the likelihood of future breast cancer risk for the next 5 years after the current screening visit (index mammogram).^22^ Dynamic prediction is a statistical methodology referring to the use of repeated measurements of predictors to estimate coefficients that link longitudinal predictors to an outcome.^22,23^ The only input required for this model is the 4 standard views of digital mammograms. The model includes prior mammograms within a specified timeframe leading up to the current screening visit. The model generates a dynamic mammogram risk score (MRS) for each woman based on changes in mammographic texture. An MRS can also be generated on a baseline mammogram without prior mammograms (Figure 1). Because the British Columbia Breast Screening Program first used digital mammograms in 2013 with biennial screening, we used a maximum of up to 4 years of prior screening mammograms to forecast a 5-year future risk.

The performance of the dynamic MRS model was assessed in terms of discrimination, calibration, and risk stratification.^24^ We used the time-dependent AUROC (5-year AUROC) in assessing the discrimination performance. The US Surveillance, Epidemiology, and End Results (SEER)–calibrated risk stratification by absolute age using dynamic MRS is reported.^25,26,27^ Additionally, we show calibration via predicted vs observed 5-year risk. The external data from the British Columbia Breast Screening Program were used solely for validation. The 95% CIs and 2-sided *P* values were estimated using 5000 bootstraps. Performance of the following models was examined: (1) age only; (2) 1-time current visit mammogram (static MRS) only; (3) up to 4 years of prior mammogram images leading up to current screening mammogram only (dynamic MRS); and (4) models 2 and 3 with age. Stratified subanalyses by race and ethnicity, including Asian (separately for South Asian and East Asian), Indigenous, and non-Hispanic White women (no subanalysis was performed for Black women due to small sample size); age (≤50 and >50 years), density (dense vs nondense), family history, and ductal carcinoma in situ (DCIS) or invasive breast cancer are reported. Statistical analysis was performed from May to August 2024 with R software version 4.4.3 (R Project for Statistical Computing).

## Results

### Cohort Characteristics

The distribution of clinical characteristics for British Columbia validation cohort is presented in Table 1. The analytic cohort of 206 929 women (mean [SD] age, 56.1 [9.7 years]) with 4 standard view FFDM mammograms, generated 4168 pathology-confirmed cases of breast cancers (883 DCIS [21.2%]), diagnosed 6 or more months after initial digital screening mammogram, during a mean (SD) follow-up time of 5.3 (3.0) years. Mean (SD) age at entry was 58.0 (9.3) years for women who developed breast cancer during follow-up, which was slightly higher than the mean (SD) age for the 202 761 individuals who remained cancer free (56.1 [9.7] years). The mean (SD) body mass index (calculated as weight in kilograms divided by height in meters squared) was higher in women who developed breast cancer (26.1 [5.5]) compared with women who remained cancer free (25.6 [5.7]). The distribution of Breast Imaging Reporting and Data System (BI-RADS) breast density was comparable between the groups, although not routinely recorded at all screening sites. Not all screening clinics recorded race or ethnicity. For those that did (118 093 women), the majority of women were White (66 742 women [56.5%]), 34 266 (29.0%) were East Asian, 6116 (5.2%) were South Asian, and 1946 (1.6%) were Indigenous women. Of all cancer-free participants, 33 073 (16.3%) had a positive family history of breast cancer (sister, mother, or both), compared with 1099 (26.4%) among those with pathology-confirmed cases of breast cancers.

**Table 1.** Baseline Characteristics by Subsequent Breast Cancer Case Status (External Validation of the British Columbia Breast Screening Program)

Characteristic	British Columbia validation cohort, No. (%) (N = 206 929)	
	Breast cancer (n = 4168)	Cancer-free cohort (n = 202 761)
Age, mean (SD) [IQR], y	58.0 (9.3) [50.7-65.2]	56.1 (9.7) [48.2-63.3]
Body mass index, mean (SD) [IQR]^a^	26.1 (5.5) [22.5-28.7]	25.6 (5.7) [21.9-28.1]
No. of longitudinal mammograms, mean (SD) [IQR]	2.3 (1.4) [1-3]	2.5 (1.3) [1-3]
No. of years between mammograms, mean (SD) [IQR]	1.9 (0.7) [1.1-2.1]	2.0 (0.7) [1.5-2.1]
Breast Imaging Reporting and Data System breast density		
A (mostly fatty)	478 (11.5)	30091 (14.8)
B (scattered fibroglandular density)	910 (21.8)	48934 (24.1)
C (heterogeneously dense)	1048 (25.1)	50391 (24.9)
D (extremely dense)	243 (5.8)	12471 (6.2)
Not recorded	1487 (35.8)	60859 (30.0)
Family history of breast cancer (mother, sister, or both)	1099 (26.4)	33073 (16.3)
Race and ethnicity		
Asian		
East Asian	786 (18.9)	33480 (16.5)
South Asian	122 (2.9)	5994 (3.0)
Black	10 (0.2)	511 (0.3)
Indigenous women	39 (0.9)	1907 (0.9)
White	1556 (37.4)	65186 (32.2)
Remaining, not listed above	207 (5.0)	8403 (4.1)
Not reported	1556 (34.7)	87280 (43.0)
Time to cancer diagnosis from first full field digital mammography		
6 mo-5 y	2540 (46.0)	NA
>5 y	1628 (29.5)	NA
Ductal carcinoma in situ	883 (21.2)	NA

Abbreviation: NA, not applicable.

^a^ Calculated as weight in kilograms divided by height in meters squared.

On average, the cohort had a mean (SD) of 1.8 (0.9) mammograms in the 4 years prior to the current clinic visit (Table 2). The mean (SD) number of years between mammograms was 2.0 (0.7) in cancer-free women, reflecting the biennial screening program (Table 1).

**Table 2.** External Validation Prediction Performance for 5-Year Breast Cancer Risk in the British Columbia Breast Screening Program by Screening Mammogram History (N = 206 929)^a^

No. of years since current clinic visit	No. of previous mammograms, mean (SD)	5-y AUROC (95% CI)		
		Age	MRS only	MRS and age
0 (static model)	1.0 (0)	0.54 (0.52-0.56)	0.71 (0.69-0.72)	0.71 (0.69-0.72)
2 (dynamic model)	1.4 (0.6)	NA	0.75 (0.73-0.77)	0.75 (0.73-0.77)
4 (dynamic model)	1.8 (0.9)	NA	0.78 (0.77-0.80)	0.78 (0.77-0.80)

Abbreviations: AUROC, area under the receiver operating characteristic curve; MRS, mammogram risk score; NA, not applicable.

^a^ Women with a diagnosis of breast cancer within 6 months of their index mammogram are excluded.

### Prediction Accuracy of 5-Year Breast Cancer Risk Using Prior Mammograms

We first assessed the performance of the baseline model at cohort entry clinic visit, using only age, and observed a 5-year AUROC of 0.54 (95% CI, 0.52-0.56). Next, we fit the model using images from the index mammogram (MRS) at screening visit (static model) and observed a 5-year AUROC of 0.71 (95% CI, 0.69-0.72). The AUROC remained unchanged when age was added to the MRS (Table 2).

We then evaluated performance using the mammogram at the current screening visit with the addition of up to 4 prior years of screening mammograms (dynamic). With the addition of the previous 4 years of screening mammograms (dynamic MRS), without age, the 5-year AUROC was 0.78 (95% CI, 0.77-0.80) (Table 2). Including age did not change the AUROC. Summarizing change in images over time, when available, significantly improved the prediction accuracy compared with using only the MRS from the index mammogram (AUROC, 0.78; 95% CI, 0.77-0.80 vs 0.71; 95% CI, 0.69-0.72; *P* < .001).

### Sensitivity and Subgroups Analysis

We compared varying numbers of prior mammograms and prediction performance. We observed an increasing AUROC for 5-year risk, from 0.71 (95% CI, 0.69-0.72) with only the current (index) screening mammogram to 0.75 (95% CI, 0.73-0.77) when including up to 2 years of prior images, and 0.78 (95% CI, 0.77-0.80) with up to 4 years of images. Using up to 4 prior years, the mean (SD) number of mammogram examinations included in the 5-year prediction was 1.8 (0.9) (Table 2).

We next assessed performance across subgroups of the screened population (eTable 1 in Supplement 1). Model performance was consistent across Asian and Indigenous women. The AUROC for White women was 0.80 (95% CI, 0.78-0.82), the AUROC for East Asian women was 0.77 (95% CI, 0.75-0.79), the AUROC for South Asian women was 0.75 (95% CI, 0.71-0.79), and the AUROC for Indigenous women was 0.77 (95% CI, 0.71-0.83). There were no statistically significant differences between White women and women of other race and ethnicities (*P* > .05). Among women aged 50 years or younger, the AUROC was 0.76 (95% CI ,0.74-0.78) and for those older than 50 years, it was 0.80 (95% CI, 0.78-0.82). Consistent with this age finding, the AUROC among women with dense breasts (BI-RADS C and D) was 0.77 (95% CI, 0.75-0.79) and for those with nondense breasts, the AUROC was 0.80 (95% CI, 0.78-0.82). Analysis separately for risk of DCIS (AUROC, 0.78; 95% CI, 0.76-0.81) and invasive disease (AUROC, 0.79; 95% CI, 0.77-0.81) showed equivalent performance. Results were largely unchanged for women with a family history of breast cancer (5-year AUROC, 0.76; 95% CI, 0.73-0.78) and without family history (AUROC, 0.78; 95% CI, 0.76-0.81).

### Dynamic MRS and Risk Calibration to SEER

The dynamic MRS was calibrated to population incidence using the 2016 US SEER 5-year expected incidence.^25^ We next applied cut points that correspond to the National Comprehensive Cancer Network (NCCN)^28^ high-risk cutoff of 1.7% 5-year risk and the American Society of Clinical Oncology (ASCO)^29^ and USPSTF^30^ cutoff of 3% 5-year risk.

**Figure 2.**
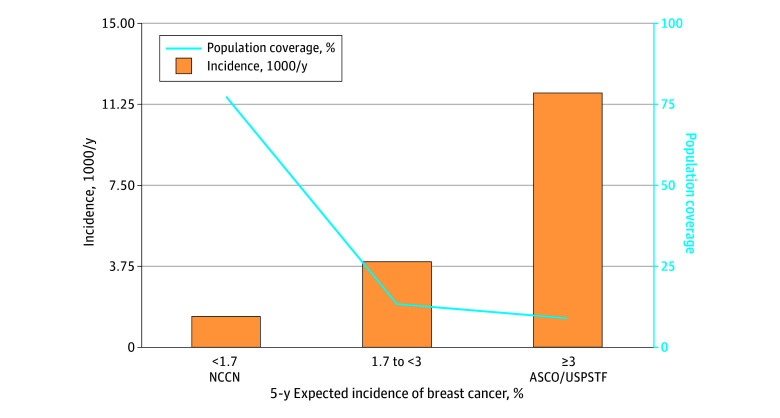
Distribution of Incidence Per 1000 Person-Years by National Comprehensive Cancer Network (NCCN), American Society of Clinical Oncology (ASCO), and US Preventive Services Task Force (USPSTF) Risk Cut Points Applied to 5-Year Risk in British Columbia Orange bars denote incidence after excluding cases diagnosed within 6 months of entry to cohort.

When using NCCN cutoff, 22.5% of the population (46 662 participants) was classified as high risk and the positive predictive value was 3.0% (incidence, 7 per 1000 person-years) (Figure 2). Using the ASCO 3% cutoff, 9.0% of the population (18 839 participants) was in the high-risk category; the positive predictive value was 4.9% (incidence, 11.8 per 1000 person-years).

We further illustrate the distribution of the dynamic MRS in eFigure 2 in Supplement 1. This plot showed good separation between women who developed breast cancer and those who remained cancer-free, consistent with the observed AUROC.

**Figure 3.**
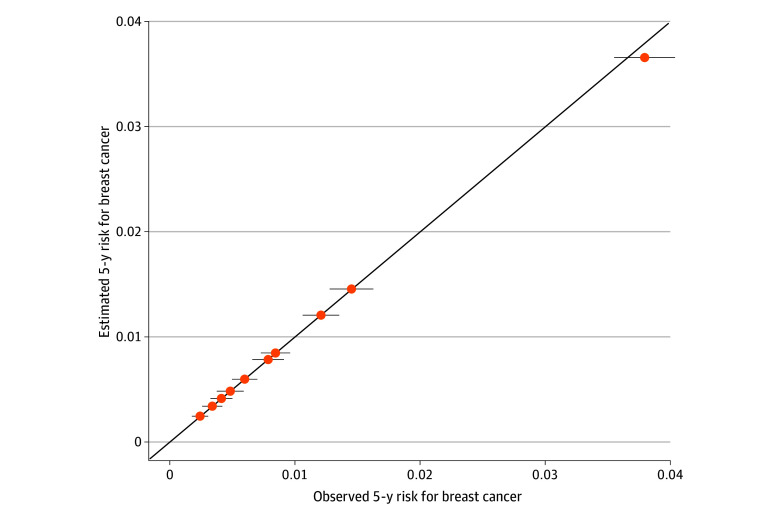
Calibration Plot of Observed vs Predicted 5-Year Breast Cancer Risk With Observed 95% CI in the British Columbia Breast Screening Program by Decile of Predicted Risk

Finally, we present predicted vs observed 5-year risk by decile with 95% CIs in Figure 3 and the corresponding counts by deciles in eTable 2 in Supplement 1. These data showed good calibration across all levels of risk. We did not observe any significant overestimation or underestimation of risk across all deciles of risk.

## Discussion

In this prognostic study, we used a dynamic risk prediction model that incorporates multiple previous mammogram images, when available, to account for subclinical changes in breast tissue over time.^9,10^ Predicted risk of breast cancer obtaining a 5-year AUROC of 0.78 (95% CI, 0.77 – 0.80) was based on analysis of screening mammogram images alone. Unlike other AI-based risk prediction models that rely only on a single mammogram, such as Mirai,^3^ our model can perform risk prediction using either a single mammogram or multiple prior mammogram images. Changes in a woman’s mammogram over time can lead to more accurate risk prediction; these changes rely on AI-based models to see changes before they are evident to the human eye. This model was previously validated in Black and White women,^10^ and here we observe robust performance when applied in different racial and ethnic groups in an organized population-based screening program. The long running British Columbia Breast Screening Program began using digital mammography in 2013. This organized program covers a population with risk distribution comparable to that of women being screened in the UK^31^ and is distinct from the opportunistic screening services in the US. The timeframe for the development and external datasets with digital screening mammograms and follow-up for incident pathology confirmed breast cancers are comparable. Women start screening every 2 years from age 40 years until age 74 years.^15^ Only a very small percentage of women positive for *BRCA* are referred to magnetic resonance imaging screening.^31^ Drawing on data from more than 200 000 women, we observed consistent performance by age and across racial and ethnic groups including South and East Asian women and Indigenous women, with an overall AUROC for 5-year risk of 0.78. The timeframe for this external cohort evaluation included cases diagnosed through 2023. Importantly, there was no change in early- or late-stage breast cancer across calendar time, suggesting limited impact of the COVID-19 pandemic on cancer detection. Additionally, while participation in screening has been reported to vary by race and ethnicity,^32^ within this screening cohort, the frequency of repeated screening was consistent across groups.

This model reflects an improvement in both accuracy and generalizability across racial and ethnic groups from AI-based and clinical factor–based breast cancer risk prediction models that use only 1 baseline mammogram to predict long-term future risk. Those models have shown modest performance for 5-year AUROC from 0.63 to 0.67^5^ and are largely trained on White women.^11^ Our dynamic risk prediction model that was trained and validated on Black and White women in the US^10^ shows consistent, robust high performance across racial and ethnic groups in an independent population with a traditional screening program structure. Importantly, this model can be calibrated to national cancer incidence rates and therefore allows stratification of risk into both high- and low-risk categories, bringing us closer to clinical use for precision prevention in broader, more diverse populations.

Whole mammogram images convey substantial information beyond mammographic breast density and what can be appreciated by a human eye,^33^ and they summarize additional texture features that are related to breast cancer risk independent of density.^3,34^ Advances in AI methods using a baseline mammogram^5,35^ show comparable performance to models that incorporate clinical factors plus mammographic density and polygenic risk scores.^13,36,37,38,39^ Importantly for routine screening, the AI models have shown no gain in performance with addition of clinical risk factors because this information is summarized in the structure of breast tissue.^40,41^ Likewise because mammographic breast density is already incorporated into the mammogram-based MRS,^42^ addition of density as a separate risk factor does not improve performance.

The use of an MRS, rather than the collection of clinical risk factors, is important for implementation of risk assessment on a broad scale. Current risk prediction models based on clinical risk factors require substantial use of resources from both the patient and health care clinicians, including intensive questionnaires, knowledge of personal and family health history, access to a formal risk calculation program, and additional testing for polygenic risk score estimation. In addition to the improved accuracy of the MRS, the improvement in resource utilization has the potential to make risk assessment feasible and accessible for many more women.

Mammographic screening is used for early detection in high-income countries and increasingly in middle-income countries as the global burden of breast cancer increases.^43^ Our results support greater use of available mammogram data from an individual’s current and previous images to improve personal risk assessment and speed the delivery of established risk-based prevention and screening.^4,44,45^ Development of the dynamic MRS and calibration to SEER facilitates direct application to existing risk management guidelines and calibration to country-specific incidence. Efficient calculation of breast cancer risk based on validated tools, developed and validated in diverse populations, is essential to achieve applicability and reduce risk of bias.^46^ Here, we bridge an important gap by using screening program data covering a diverse population different from the model development and validation.

Improved risk classification may also help build trust in mammography. In the UK, participation is approximately 62% and in British Columbia approximately 50% of invited women participate in regular screening.^15^ For Europe as a whole among women aged 50 to 69 years, 79% are invited to screening visits and 49% participate.^47^ Meanwhile, in the US, 68% of women reported having had mammogram within recommended screening interval (2018).^48^ Increased accuracy and confidence in an individually tailored screening program may encourage more eligible women to participate.^49^

### Strengths and Limitations

Our study has several strengths. Unlike other breast cancer risk prediction models, ours includes images from prior screenings, made possible by the expansion of digital imaging. Here, we offer a validated dynamic risk prediction model to analyze repeated mammograms efficiently and demonstrate its utility in a racially and ethnically diverse external validation dataset. It can accommodate varying number of mammograms (1 or more) and time intervals between screening as applicable in different countries and using different starting ages for screening. Our model is calibrated to SEER and allows calibration to other population incidence rates. Hence, it can apply to country-specific screening programs and policies.

This study also has limitations. We constrained our analysis to utilize mammograms from the prior 4 years up to the current visit due to the maximum years of follow-up after implementing digital imaging in 2013. The potential improvement with more prior mammograms remains to be explored. Whether specific risk factors or addition of polygenic risk scores add to performance despite added participant burden also requires study. Here, we used a 5-year time horizon because this is the initial US Food and Drug Administration indication for tamoxifen for chemoprevention^50^ and for subsequent US guidelines from NCCN and ASCO for risk classification and breast risk management.^28,29^

## Conclusion

Given the global burden of breast cancer among women, and the range of screening intervals and starting ages in screening programs,^47^ it is imperative to use existing image data to more accurately assess risk to guide precision prevention and maximize population benefits.^4,51^ The dynamic risk prediction model used in this prognostic study makes full use of each woman’s own mammograms for personalized long-term risk prediction. It performed equally well across a diverse range of racial and ethnic groups and in a different screening setting. These findings indicate that adding prior mammograms significantly improves 5-year risk prediction.

## Notes

Supplement 1. **eFigure 1.** British Columbia Breast Screening Program Cohort **eTable 1.** Five-Year Breast Cancer Risk Prediction Performance in British Columbia Breast Screening Program for Subgroups Using the Dynamic MRS With up to Past 4 Years of Mammograms, Represented by AUC and 95% Confidence Interval **eFigure 2.** Plot of Dynamic MRS at Prediction for Women Who Subsequently Developed Breast Cancer (Red) and Those Who Remained Free From Breast Cancer (Blue) During 5 Years of Follow-Up **eTable 2.** 5-Year Risk of Breast Cancer Across the Deciles of Predicted Risk in the British Columbia Breast Screening External Validation Using up to 4 Years of FFDM Mammograms

Supplement 2.
Data Sharing Statement
