# Supplementary material for: Validation of a Dynamic Risk Prediction Model Incorporating Prior Mammograms in a Diverse Population
Source: JAMA Netw Open. 2025 Jun 6;8(6):e2512681. doi: 10.1001/jamanetworkopen.2025.12681 (PMC12144620; doi:10.1001/jamanetworkopen.2025.12681)
Supplement: Supplement 1. — eFigure 1. British Columbia Breast Screening Program Cohort eTable 1. Five-Year Breast Cancer Risk Prediction Performance in British Columbia Breast Screening Program for Subgroups Using the Dynamic MRS With up to Past 4 Years of Mammograms, Represented by AUC and 95% Confidence Interval eFigure 2. Plot of Dynamic MRS at Prediction for Women Who Subsequently Developed Breast Cancer (Red) and Those Who Remained Free From Breast Cancer (Blue) During 5 Years of Follow-Up eTable 2. 5-Year Risk of Breast Cancer Across the Deciles of Predicted Risk in the British Columbia Breast Screening External Validation Using up to 4 Years of FFDM Mammograms [file jamanetwopen-e2512681-s001.pdf]

Supplemental Online Content

Jiang S, Bennett DL, Colditz GA. Validation of a dynamic risk prediction model incorporating prior mammograms in a diverse population. *JAMA Netw. Open*. 2025;8(6):e2512681. doi:10.1001/jamanetworkopen.2025.12681

eFigure 1. British Columbia Breast Screening Program Cohort
eTable 1. Five-Year Breast Cancer Risk Prediction Performance in British Columbia Breast Screening Program for Subgroups Using the Dynamic MRS With up to Past 4 Years of Mammograms, Represented by AUC and 95% Confidence Interval
eFigure 2. Plot of Dynamic MRS at Prediction for Women Who Subsequently Developed Breast Cancer (Red) and Those Who Remained Free From Breast Cancer (Blue) During 5 Years of Follow-Up
eTable 2. 5-Year Risk of Breast Cancer Across the Deciles of Predicted Risk in the British Columbia Breast Screening External Validation Using up to 4 Years of FFDM Mammograms

This supplemental material has been provided by the authors to give readers additional information about their work.

© 2025 Jiang et al. *JAMA Network Open.*

eFigure 1
British Columbia Breast Screening Program Cohort

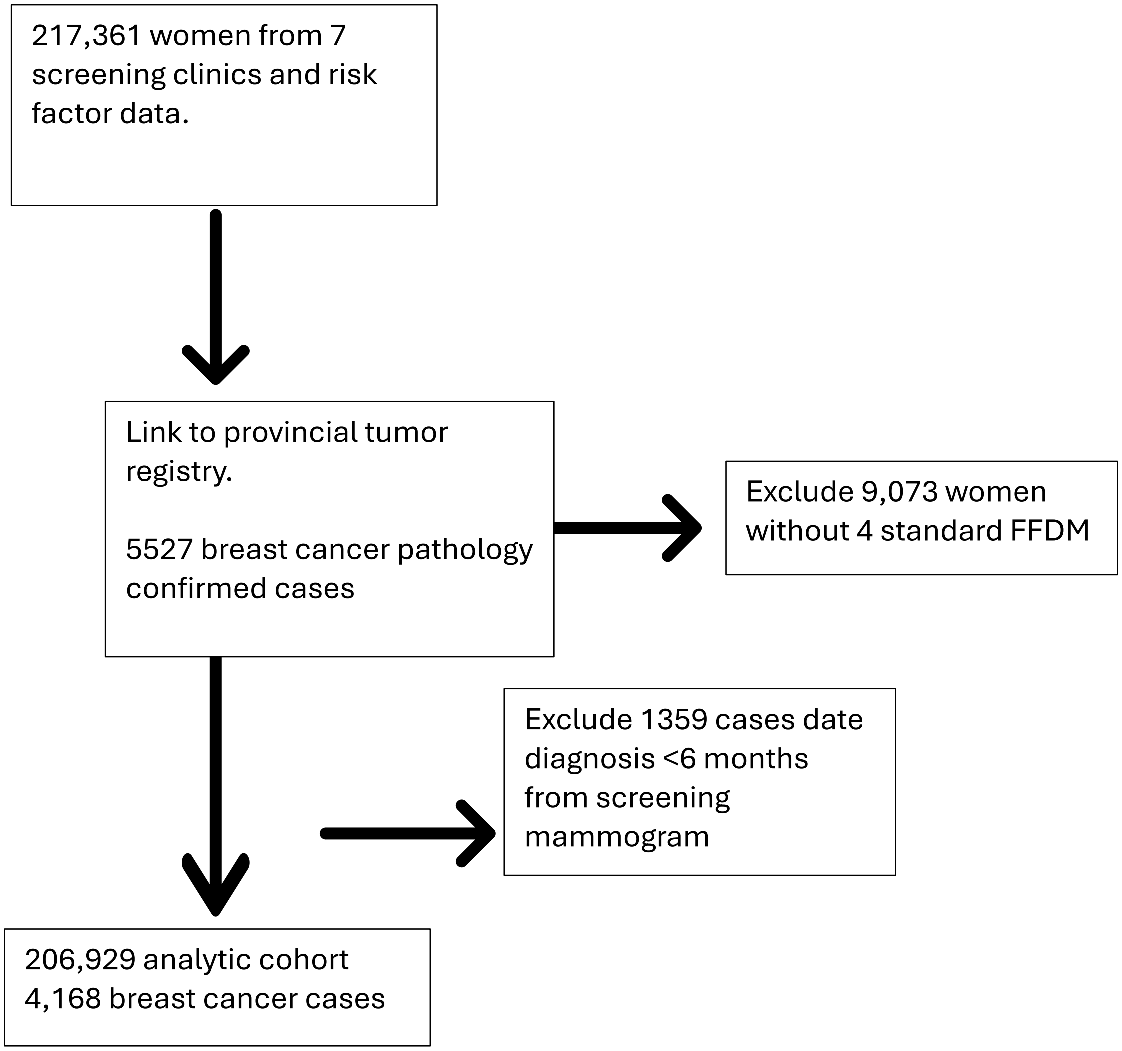

© 2025 Jiang et al. *JAMA Network Open*.

eTable 1. Five-year breast cancer risk prediction performance in British Columbia breast screening program for subgroups using the dynamic MRS with up to past 4 years of mammograms, represented by AUC and 95% Confidence Interval.

Subgroup	External validation cohort (British Columbia)
	5-year AUC (95%CI)
**Race-ethnicity**	
Non Hispanic White	0.80 (0.78, 0.82)
Non Hispanic Black	--
Asian	0.77 (0.75, 0.79)
a. East Asian	0.77 (0.75, 0.79)
b. South Asian	0.75 (0.71, 0.79)
Indigenous Women	0.77 (0.71, 0.83)
**BIRADS breast density**	
Nondense (A / B)	0.80 (0.78, 0.82)
Dense (C / D)	0.77 (0.75, 0.79)
**Age**	
≤50 years	0.76 (0.74, 0.78)
>50 years	0.80 (0.78, 0.82)
**Cancer subtype**	
DCIS	0.78 (0.76, 0.81)
Invasive	0.79 (0.77, 0.81)
**Family History**	
Yes	0.76 (0.73, 0.78)
No	0.78 (0.76, 0.81)

Women with a diagnosis of breast cancer within 6 months of their index mammogram are excluded.

© 2025 Jiang et al. *JAMA Network Open*.

eFigure 2. Plot of dynamic MRS at prediction for women who subsequently developed breast cancer (red) and those who remained free form breast cancer (blue) during 5 years of follow-up.

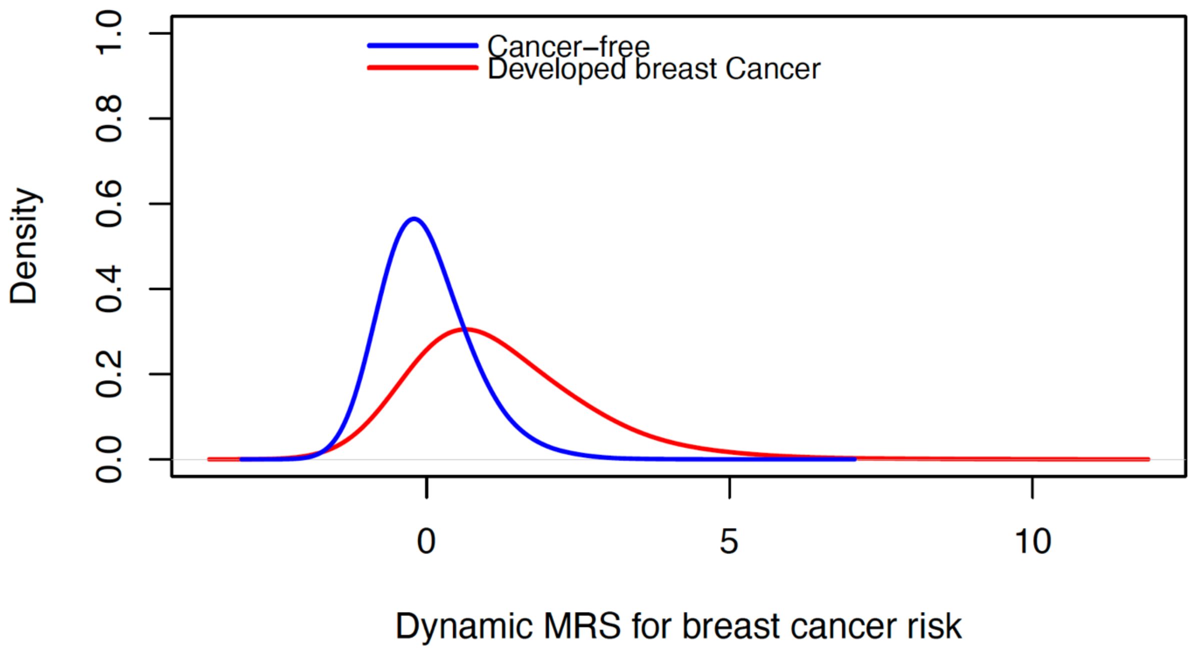

© 2025 Jiang et al. *JAMA Network Open*.

eTable 2. 5-year risk of breast cancer across the deciles of predicted risk in the British Columbia breast screening external validation using up to 4 years of FFDM mammograms. Predicted risk is ordered from Q1 (lowest) to Q10 (highest), and equal number of participants were assigned in each decile if possible.

Decile predicted risk	Observed 5-year risk (%, 95% CI)	Predicted 5-year risk (%)
Q1 (n = 20,693)	0.16 (0.10, 0.21)	0.16
Q2 (n = 20,693)	0.27 (0.21, 0.34)	0.27
Q3 (n = 20,693)	0.35 (0.26, 0.43)	0.35
Q4 (n = 20,693)	0.49 (0.39, 0.58)	0.48
Q5 (n = 20,693)	0.61 (0.50, 0.73)	0.62
Q6 (n = 20,693)	0.73 (0.62, 0.84)	0.74
Q7 (n = 20,693)	0.89 (0.77, 1.02)	0.89
Q8 (n = 20,693)	1.15 (0.99, 1.31)	1.16
Q9 (n = 20,693)	1.37 (1.19, 1.55)	1.39
Q10 (n = 20,692)	3.79 (3.54, 4.04)	3.71

© 2025 Jiang et al. *JAMA Network Open*.
