# Supplementary material for: Validation of a Dynamic Risk Prediction Model Incorporating Prior Mammograms in a Diverse Population
Source: JAMA Netw Open. 2025 Jun 6;8(6):e2512681. doi: 10.1001/jamanetworkopen.2025.12681 (PMC12144620; doi:10.1001/jamanetworkopen.2025.12681)
Supplement: Supplement 2. — Data Sharing Statement [file jamanetwopen-e2512681-s002.pdf]

Data Sharing Statement

Jiang. Validation of a Dynamic Risk Prediction Model Incorporating Prior Mammograms in a Diverse Population. *JAMA Netw Open*. Published June 06, 2025. doi:10.1001/jamanetworkopen.2025.12681

Data
Data available: No

Additional Information
Explanation for why data not available: data are the property of the province of British Columbia. The data are the property of BC Cancer. Deidentified data are accessed through a Research Ethics Board approved protocol to achieve the aims of the approved proposal. Interested users can contact Rasika Rajapakshe (RRajapak at bccancer.bc.ca). Data requestors will need to sign a data access agreement.
